# Lrig1 regulates the balance between proliferation and quiescence in glioblastoma stem cells

**DOI:** 10.3389/fcell.2022.983097

**Published:** 2022-10-26

**Authors:** Kirsty M. Ferguson, Carla Blin, Neza Alfazema, Ester Gangoso, Steven M. Pollard, Maria Angeles Marques-Torrejon

**Affiliations:** ^1^ Centre for Regenerative Medicine and Edinburgh Cancer Research UK Centre, Institute for Regeneration and Repair, University of Edinburgh, Edinburgh, United Kingdom; ^2^ Predepartment Unit of Medicine. Jaume I University, Castellon, Spain

**Keywords:** glioblastoma, neural stem cell, quiescence, Lrig1, BMP

## Abstract

Patients with glioblastoma (GBM) face a dismal prognosis. GBMs are driven by glioblastoma stem cells (GSCs) that display a neural stem cell (NSC)-like phenotype. These glioblastoma stem cells are often in a quiescent state that evades current therapies, namely debulking surgery and chemo/radiotherapy. Leucine-rich repeats and immunoglobulin-like domains (LRIG) proteins have been implicated as regulators of growth factor signalling across many tissue stem cells. Lrig1 is highly expressed in gliomas and importantly, polymorphisms have been identified that are risk alleles for patients with GBM, which suggests some functional role in gliomagenesis. We previously reported that Lrig1 is a gatekeeper of quiescence exit in adult mouse neural stem cells, suppressing epidermal growth factor receptor signalling prior to cell cycle re-entry. Here, we perform gain- and loss-of-function studies to understand the function of Lrig1 in glioblastoma stem cells. Using a novel mouse glioblastoma stem cell model, we show that genetic ablation of *Lrig1* in cultured GBM stem cells results in higher proliferation and loss of quiescence. *In vivo*, mice transplanted with glioblastoma stem cells lacking Lrig1 display lower survival compared to Lrig1 WT glioblastoma stem cells, with tumours displaying increased proportions of proliferative cells and reduced quiescent subpopulations. In contrast, Lrig1 overexpression in mouse glioblastoma stem cells results in enhanced quiescence and reduced proliferation, with impaired tumour formation upon orthotopic transplantation. Mechanistically, we find that Lrig1-null cells have a deficiency in BMP signalling responses that may underlie their lack of responsiveness to quiescence cues *in vivo*. These findings highlight important roles for Lrig1 in controlling responsiveness to both epidermal growth factor receptor and BMPR signalling, and hence the proportions of quiescent and proliferative subpopulations in GBMs.

## Introduction

Glioblastoma multiforme (GBM) is the most common and aggressive primary adult brain tumour. Standard treatment involves surgical resection of the tumour mass followed by adjuvant radiotherapy and chemotherapy (temozolomide; TMZ) (Stupp et al., 2005; Arbab et al., 2017; Fernandes et al., 2017; Villa et al., 2018; Wen et al., 2020; Khan et al., 2021). However, this is rarely curative, and only 5.5% of patients survive 5 years following diagnosis. For the vast majority of patients, tumours regrow from residual disease in the 1–2 cm margin of the resection cavity. These poor survival rates in GBM are likely in part due to sub-populations of quiescent stem cell-like cells, termed cancer stem cells (CSCs), that escape current therapies.

CSCs have the ability to both self-renew and produce heterogeneous cancer cells that comprise the tumour bulk, thus driving tumorigenesis (Kreso and Dick, 2014; Nassar and Blanpain, 2016; Batlle and Clevers, 2017). In GBM, neural stem cell (NSC)-like cells (GBM stem cells; GSCs) drive tumour progression (Galli et al., 2004; Singh et al., 2004; Beier et al., 2007; Ogden et al., 2008; Son et al., 2009; Lathia et al., 2010; Chen et al., 2012; Haas et al., 2017). Similar to normal adult NSCs, GSCs can likely adopt a continuum of states, from quiescence (deep or shallow) to active proliferation. Quiescent GSCs are able to evade anti-mitotic therapies through their reversible cell-cycle arrest (Deleyrolle et al., 2011; Chen et al., 2012). This selective survival is likely a major factor leading to tumour recurrence. It is therefore crucial for us to understand the molecular regulation of the balance between proliferation and quiescence in GSCs to improve therapies.

Low-grade gliomas express higher levels of BMP4 than high-grade gliomas, correlating to lower mortality rates; this suggests BMP could be a prognostic marker in gliomas (Bao et al., 2013). BMP signalling induces cell-cycle exit and quiescence of normal NSCs (Bonaguidi et al., 2005; Piccirillo et al., 2006; Mira et al., 2010; Marqués-Torrejón et al., 2021). Indeed, exposure of patient-derived GSCs to BMP4 can drive astrocyte differentiation both *in vitro* and *in vivo* (Gross et al., 1996; Piccirillo et al., 2006; Carén et al., 2015), raising the possibility that this pathway could be exploited for differentiation therapy. However, terminal BMP-driven differentiation of GBM is challenging, as GSCs fail to undergo epigenetic progression and are able to revert upon BMP withdrawal (Carén et al., 2015). This might be due to partial differentiation and then phenotypic plasticity; however, our recent studies suggest that BMP in combination with RTK signalling induces a quiescent GSC state rather than terminal differentiation (Marqués-Torrejón et al., 2021), consistent with its role in adult neural stem cell niches. Understanding the factors limiting the cytostatic effects of BMP may help to improve therapeutic outcomes.

Deregulated growth factor signalling is a major driver of GBM initiation and progression. Leucine-rich repeats and immunoglobulin-like domains (LRIG) proteins are well-known regulators of growth factor signalling (Mao et al., 2017). For example, the tumour suppressor and stem cell marker Lrig1 is a negative regulator of the epidermal growth factor receptor (EGFR) family (Gur et al., 2004; Laederich et al., 2004; Powell et al., 2012). Recently we uncovered Lrig1 as a gatekeeper of quiescence in adult neural stem cells, through its roles in limiting EGFR signalling (Marqués-Torrejón et al., 2021). A decrease in Lrig1 has emerged as an indicator of worse prognosis in several cancer types (Rouam et al., 2010). Lrig1 has been shown to be more highly expressed in gliomas than other human cancers (Ji et al., 2022) and genome-wide association studies have identified a risk allele (rs11706832) associated with glioma susceptibility (Melin et al., 2017). LRIG1 is a potent inhibitor of GBM growth in clinically-relevant experimental glioma models, largely independent of EGFR status (Johansson et al., 2013) and is thought to restrict proliferation and aggressiveness of GSCs (Mao et al., 2017).

Recently, LRIG proteins have been shown to be evolutionarily conserved regulators of both lipid metabolism and BMP signalling (Hedman and Henriksson, 2007; Herdenberg et al., 2021). Thus, the presence of LRIG levels may be a requirement not only to suppress EGFR signalling, but also to support BMP signalling. Mouse NSCs can be readily transformed into GSCs by deletion or overexpression of tumour suppressors or oncogenes, using CRISPR/Cas9 technology and PiggyBac overexpression (Gangoso et al., 2021). These cells are tumour-initiating *in vivo*, providing a disease-relevant experimental model to investigate the mechanisms controlling GSC quiescence. Here, we utilise mouse GSC cultures to determine the function of Lrig1 in regulating proliferation and quiescence of GSCs. Our findings highlight important dual roles for Lrig1 in suppressing pro-proliferative EGFR signalling, while simultaneously enhancing cytostatic BMP signalling pathways.

## Results

### Lrig1 deletion in glioblastoma stem cells enhances proliferation

To determine the function of Lrig1 in GSCs, we first engineered genetically normal cultured adult mouse NSCs into tumour-initiating GSCs. To achieve this, we used previously reported adult mouse NSCs containing an improved Fucci reporter (Mort et al., 2014; Marqués-Torrejón et al., 2021). These were transformed *via* CRISPR deletion of *Trp53* (encoding P53) and PiggyBac-mediated delivery of an EGFRvIII over-expression plasmid (Supplementary Figures S1A–D). These cells, termed PE, were confirmed as fully transformed and tumour initiating by *in vivo* transplantation into immunocompromised (NSG) mice. The resulting tumours could respond to treatment with cytotoxic therapy, with TMZ abolishing Venus signal (cells in S/G2/M phase) and decreasing both Ki67 and CldU (Supplementary Figures S2A–D). PE cells therefore provide a useful glioma-initiating cell line in which we could monitor the cycling cell population using the Fucci cell cycle reporter.

**FIGURE 1:**
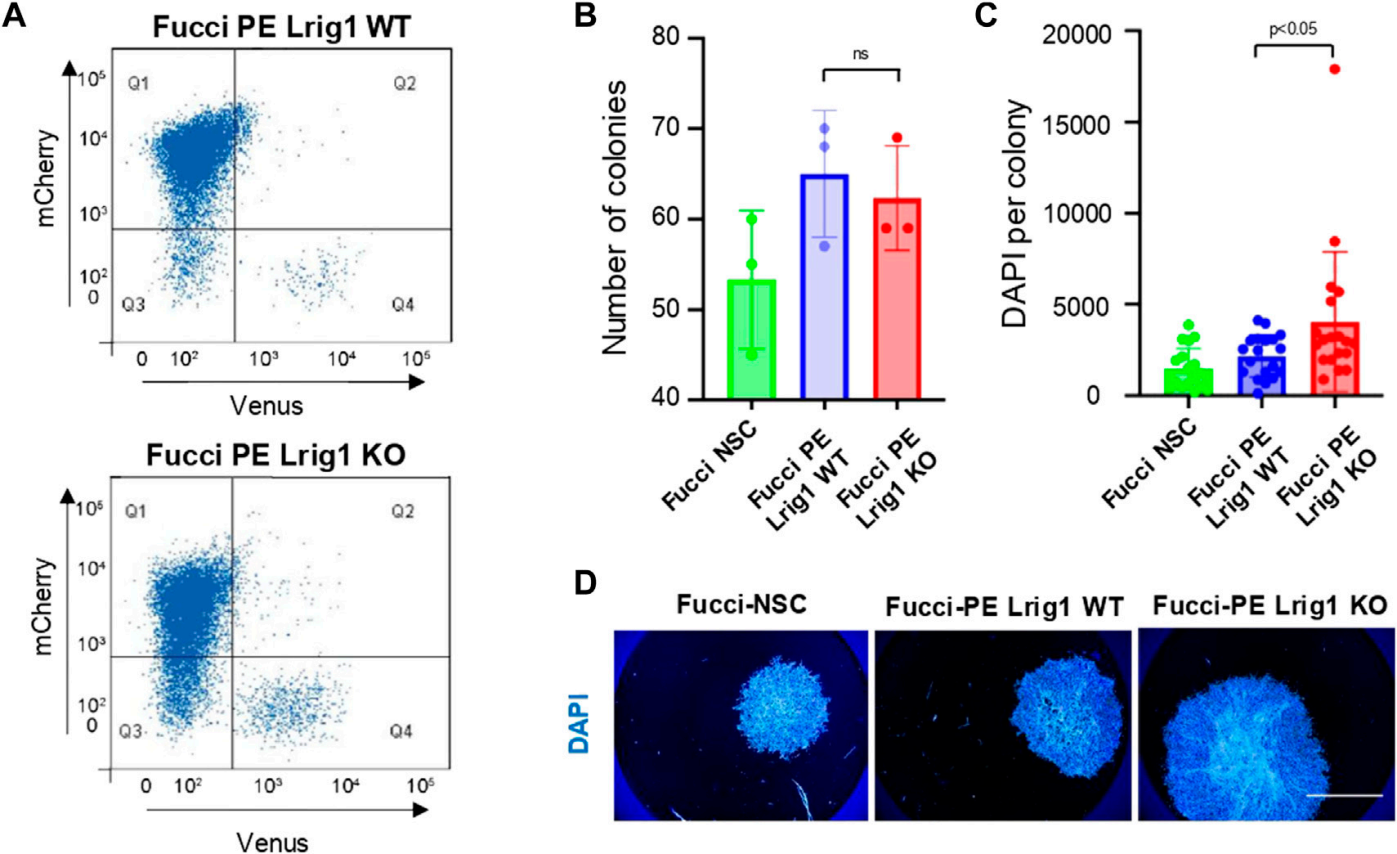
Lrig1 regulates proliferation of GSCs. **(A)** Flow analysis of Fucci mCherry-Cdt1 and aVenus-hGem reporters in Lrig1 WT and Lrig1 KO mouse GSCs. (n = 3). **(B)** Quantification of the number of colonies formed in single cell colony forming assays by Fucci NSCs, Fucci PE Lrig1 WT (GSCs with p53 KO and EGFRvIII overexpression) and Fucci PE Lrig1 KO. n = 3 (mean ± SD). **(C)** Quantification of the size of colonies formed in single cell colony forming assays by Fucci NSC, Fucci PE Lrig1 WT (GSCs with p53 KO and EGFRvIII overexpression) and Fucci PE Lrig1 KO as assessed by DAPI (n = 3 independent experiments, median ± SD, each dot represents a single colony). *p* value = 0.0456. **(D)** Representative fluorescence imaging of DAPI stained colonies using Operetta high-content analysis system. Scale bar in **(D)** is 1,500 μm.

Next, to determine Lrig1’s role in GSC proliferation we deleted *Lrig1* from PE cells using CRISPR/Cas9. Lrig1 knock-out (Lrig1-KO) cells were isolated using FACS, using a cell surface antibody to enrich for the ablated cells (Supplementary Figures S3A–C). Fucci reporter levels were assessed in the Lrig1 wild-type (WT or Lrig1^+/+^) and Lrig1 KO PE populations to determine the proportion of cells in distinct phases of the cell cycle. In WT Lrig1^+/+^ GSCs under proliferative conditions (EGF/FGF-2), a full range of cell cycle stages was observed with the Fucci reporter (Figure 1A). However, Lrig1 KO GSCs were found to display a significant increase in S/G2/M phase (Venus-positive, Q4) cells (Figure 1A), indicative of an increase in cycling cells. Consistently, using single cell colony-forming assays we found that, although colony formation by Lrig1 WT and KO cells occurred with similar frequency, Lrig1 KO colonies were on average larger in size (Figures 1B–D). Together with our previous study (Marqués-Torrejón et al., 2021), these data suggest that Lrig1 restricts proliferation of both normal NSCs and their transformed derivatives (PE-GSCs).

### Lrig1 KO glioblastoma stem cells show impaired BMP signalling

**FIGURE 2:**
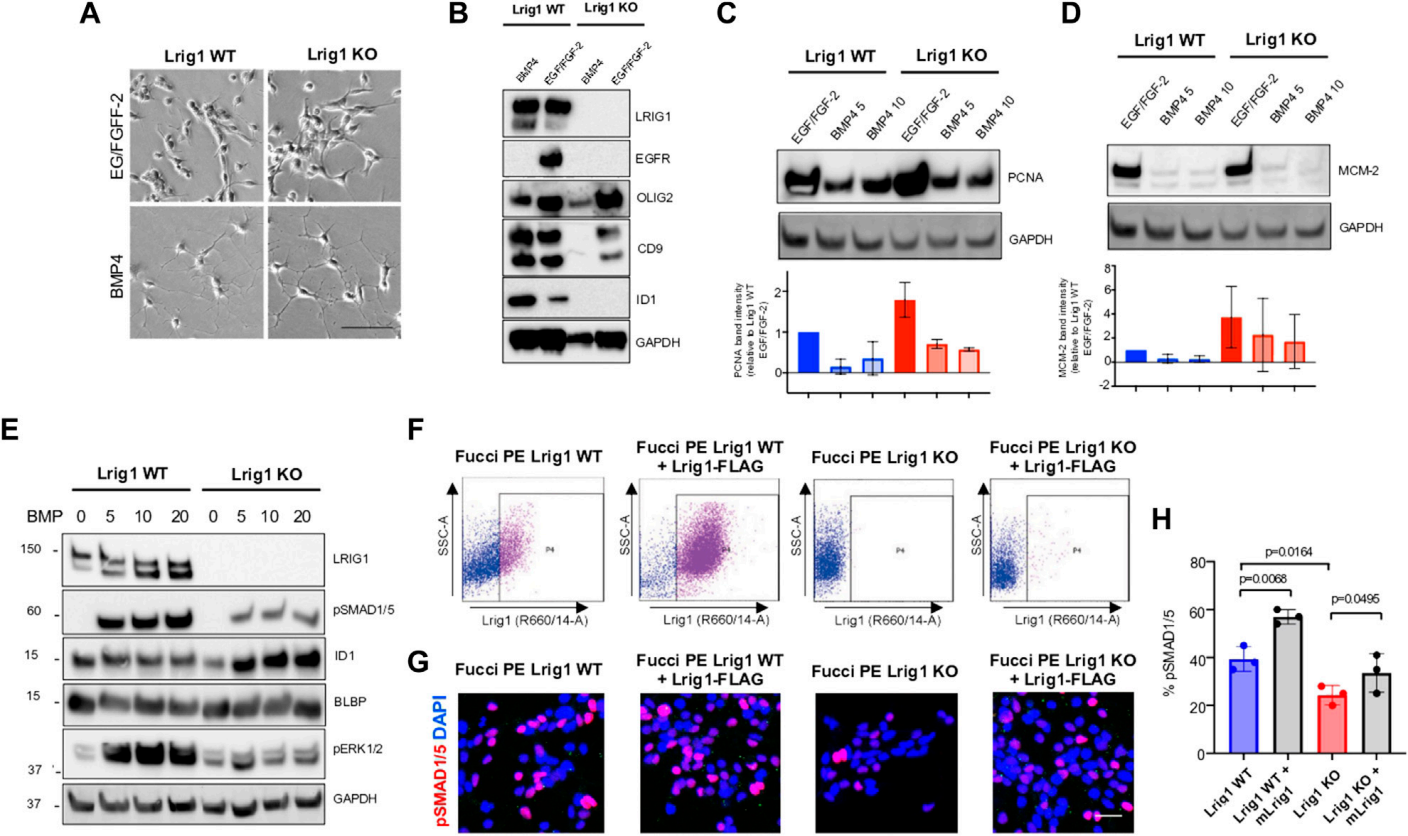
Lrig1 KO GSCs show impaired BMP signalling **(A)** Phase-contrast imaging of Lrig1 WT and Lrig1 KO GSCs treated with EGF/FGF or BMP4 for 3 days. **(B)** Immunoblot for LRIG1, EGFR, OLIG2, CD9, ID1 and GAPDH expression in Lrig1 WT and Lrig1 KO GSCs treated with EGF/FGF or BMP4 for 3 days. **(C)** Immunoblot and quantification for PCNA and GAPDH in Lrig1 WT and Lrig1 KO GSCs grown in EGF/FGF-2 or treated with 5 ng/ml or 10 ng/ml of BMP4 for 3 days. Quantification shown relative to GAPDH and Lrig1 WT EGF/FGF-2 control. **(D)** Immunoblot and quantification for MCM2 and GAPDH in Lrig1 WT and Lrig1 KO GSCs grown in EGF/FGF-2 or treated with 5 ng/ml or 10 ng/ml of BMP4 for 3 days. Quantification shown relative to GAPDH and Lrig1 WT EGF/FGF-2 control. **(E)** Immunoblot for LRIG1, pSMAD1/5, ID1, BLBP, pERK1/2 and GAPDH in Lrig1 WT and Lrig1 KO GSCs treated with different dosages of BMP4 for 3 days. Dosages in ng/ml. Protein band sizes shown in kDa. **(F)** Flow cytometry plots showing strategy to select Lrig1-positive cells in Lrig1 KO GSCs after reintroduction of mLrig1-FLAG. **(G)** Immunostaining for pSMAD1/5 (red) and nuclear counterstaining with DAPI (blue). +Lrig1-FLAG refer to the sorted populations. ICC was performed on Fucci PE Lrig1 WT, Fucci PE Lrig1 KO and the populations sorted for Lrig1-FLAG following expansion. **(H)** Quantification of the percentage of pSMAD 1/5 in the different conditions (n = 3) Unpaired two-tailed t-tests. Scale bar in **(A)** is 50 μm and **(H)** is 20 µm.

To assess Lrig1’s role in quiescence, the PE mouse GSCs were treated with replacement of growth factors with high levels of BMP4 in culture (Carén et al., 2015). Both Lrig1 WT and Lrig1 KO GSCs underwent morphological changes from a proliferative bipolar phase-bright appearance in EGF/FGF to a stellate astrocytic-like morphology upon BMP treatment (Figure 2A). Quiescence (B cell) markers, such as CD9 and Id1, and proliferative (C cell) markers, such as EGFR and Olig2, were then assessed by immunoblotting (Figure 2B). In WT Lrig1^+/+^ GSCs, BMP4 treatment reduces EGFR and OLIG2 expression and upregulates CD9 and ID1 compared to EGF/FGF (Figure 2B). Following Lrig1 KO, we still observed downregulation of the proliferation and NSC marker Olig2 upon BMP treatment. As we found previously (Marqués-Torrejón et al., 2021), endogenous EGFR protein was reduced in Lrig1 KO cells compared to Lrig1 WT, even under proliferative conditions (EGF/FGF) (we have observed this lack of EGFR band to occur in other tumour lines when the receptor is very active). Assessment of the proliferative markers, PCNA and MCM-2, expressed in all phases of the cell cycle except G0, indicated higher levels in Lrig1 KO cells than their WT counterparts in EGF/FGF-2 (Figures 2C,D). Upon BMP treatment, PCNA and MCM-2 levels decreased in both Lrig1 WT and Lrig1 KO cells. Thus, Lrig1 KO GSCs can respond to the replacement of growth factors with high levels of BMP.

To determine if the cytostatic response to BMP was dose-dependent, we next plated GSCs with different BMP doses and assessed BMP signalling by immunoblotting (Figure 2E). Interestingly, this revealed impaired BMP signalling in Lrig1 KO GSCs *via* both canonical (pSmad1/5) and non-canonical (pERK1/2) pathways (Figure 2E). Re-introduction of Lrig1 into Lrig1 KO GSCs was achieved by transfection with a mouse Lrig1 overexpression construct using the PiggyBac transposase system (FLAG-tagged mouse Lrig1 expressed from the CMV promoter) and subsequent sorting (Figure 2F). This overexpression was able to rescue the impaired BMP signalling of Lrig1 KO GSCs, as shown by rescued pSMAD1/5 levels (Figures 2G,H). Analysis of *Bmpr1A/1B/2* gene expression revealed no significant changes between Lrig1 WT and Lrig1 KO GSCs (Supplementary Figure S4), indicating a change in BMP receptor expression was not the cause of this signalling impairment, as has been observed in some human GSCs (Carén et al., 2015). Thus, mouse GSCs have increased proliferative capacity following loss of Lrig1 and display reduced responsiveness to cytostatic BMP signalling.

### Lrig1 KO reduces quiescence of BMP-treated glioblastoma stem cells and survival time of mice following glioblastoma stem cell transplantation

**FIGURE 3:**
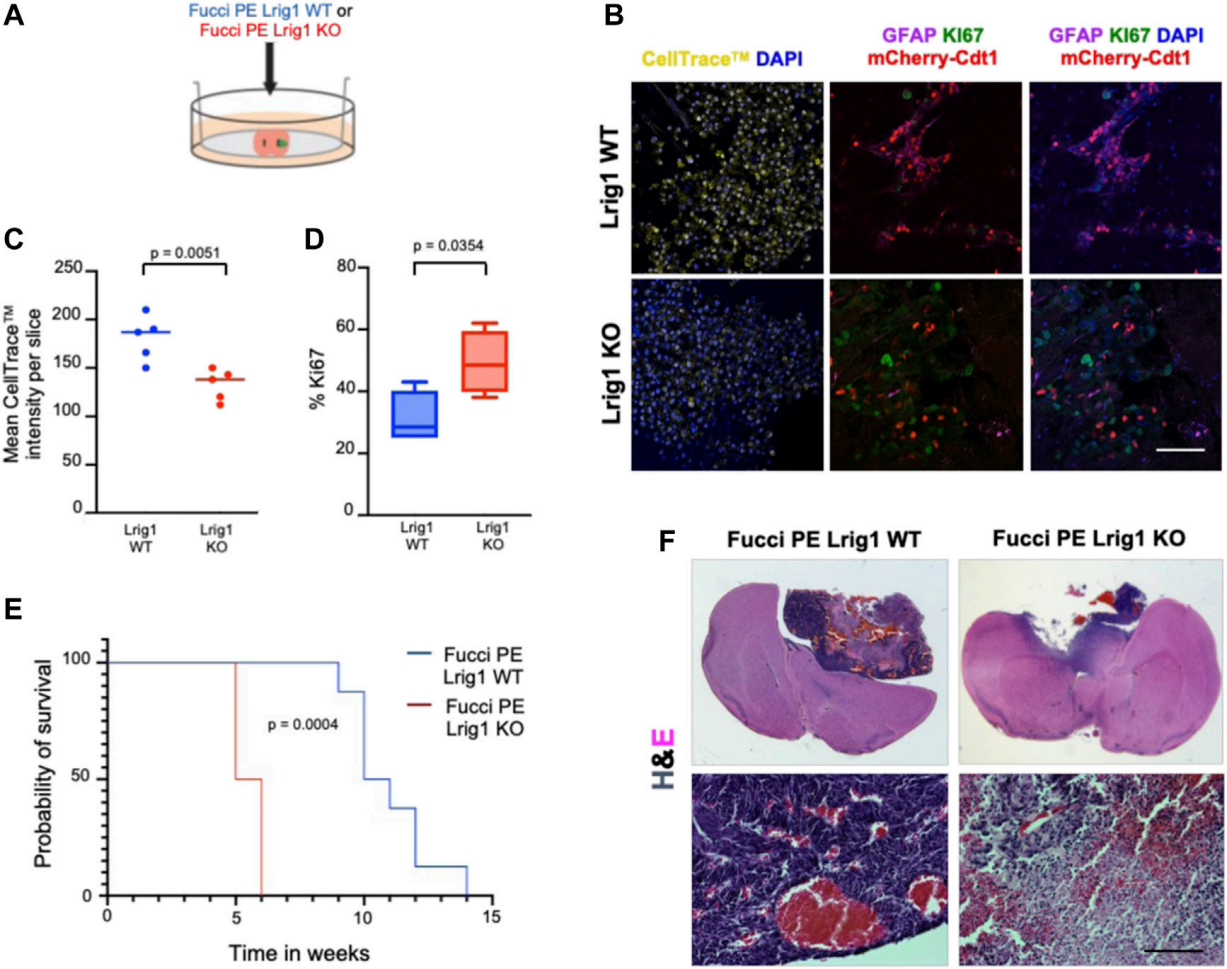
Lrig1 KO reduces quiescence features of BMP-treated GSCs and survival time of mice following GSC transplantation. **(A)** Schematic describing the microinjection of Lrig1 WT or KO GSCs into *ex vivo* organotypic brain slices. **(B)** Fluorescent CellTrace™, mCherry-Cdt1 (red) and immunofluorescence of GFAP (purple), KI67 (green) and nuclear counterstaining with DAPI (blue). Imaging following 7 days of co-culture of Lrig1 WT or KO GSCs with organotypic slice cultures (n = 5). **(C)** Quantification of the mean intensity of the CellTrace™ in the organotypic co-culture (n = 5). *p* = 0.0051. **(D)** Quantification of the percentage of KI67 positive cells in the organotypic co-culture *p* = 0.0354. **(E)** Kaplan Meier survival curve of mice with intracranial transplantation of Lrig1 WT or Lrig1 KO GSCs (n = 8 and four respectively) Log-rank (Mantel-Cox) test *p* = 0.0004. **(F)** H&E staining of coronal sections of brain tissue following co-culture with Lrig1 WT or Lrig1 KO Fucci PE GSCs for 7 days. Scale bar in **(B)** is 50 μm and **(F)** is 200 μm.

To analyse the effect of Lrig1 loss on the tumour-initiating capacity of GSCs we employed two approaches: first, *ex vivo* GSC transplantation of Lrig1 WT or Lrig1 KO GSCs into organotypic slice cultures (Marques-Torrejon et al., 2018) (Figure 3A); and second, *in vivo* transplantation into immunocompromised (NSG) mice. Using the slice culture assay we could track acute responses of GSCs to the tissue microenvironment. GSCs were microinjected into the striatum of adult mouse coronal brain slices, and after 7 days the co-culture tissue was fixed. Some GSCs expressed GFAP (astrocyte and type B cell marker) and mCherry-Cdt1 in both co-cultures. GSC proliferation was then assessed using CellTrace™ and the proliferative marker Ki67 (Figures 3B–D). Lrig1 KO GSCs showed a lower CellTrace™ intensity than Lrig1 WT cells, indicating more cell divisions had occurred during the 7 days of co-culture (diluting the CellTrace™ signal) (Figures 3B,C). Similarly, a higher percentage of Ki67 cells was found in the Lrig1 KO population (Figures 3B,D).

Following *in vivo* transplantation of either Lrig1 WT or Lrig1 KO GSCs into the striatum of NSG mice, we found that Lrig1 KO GSCs have greater tumour-initiating capacity and consequently mice showed reduced survival (Figures 3E,F). Immunostaining was performed to assess both proliferative/stem cell (Nestin, Sox2, Ki67, Vimentin, SSEA1, CD133) and quiescent (Cd9 and Gfap) markers within the tumours (Supplementary Figure S5). We detected significantly higher KI67 and significantly lower GFAP expression in the *Lrig1* KO tumours than WT controls. While not significant, higher levels of the stem cell markers Nestin, Vimentin and SSEA1 were also seen in the Lrig1 KO tumours. This confirms that Lrig1 KO enhances GSC proliferation *in vivo* and *ex vivo,* consistent with our *in vitro* cell culture data.

### Lrig1 overexpression reduces proliferation and enhances the expression of quiescence markers in glioblastoma stem cells *in vitro*

We next utilised a distinct highly aggressive GSC model cell line, termed NPE-IE (Gangoso et al., 2021). NPE-IE cells contain the common GBM mutations *Nf1* loss, *Pten* loss, and EGFRvIII overexpression. They were generated from secondary tumours following serial transplantation of NPE cells in BL6 host mice and have undergone epigenetic immunoediting to generate a highly aggressive and immune evasive (NPE-IE) GSC model reminiscent of the ‘mesenchymal’ subtype of GBM.

**FIGURE 4:**
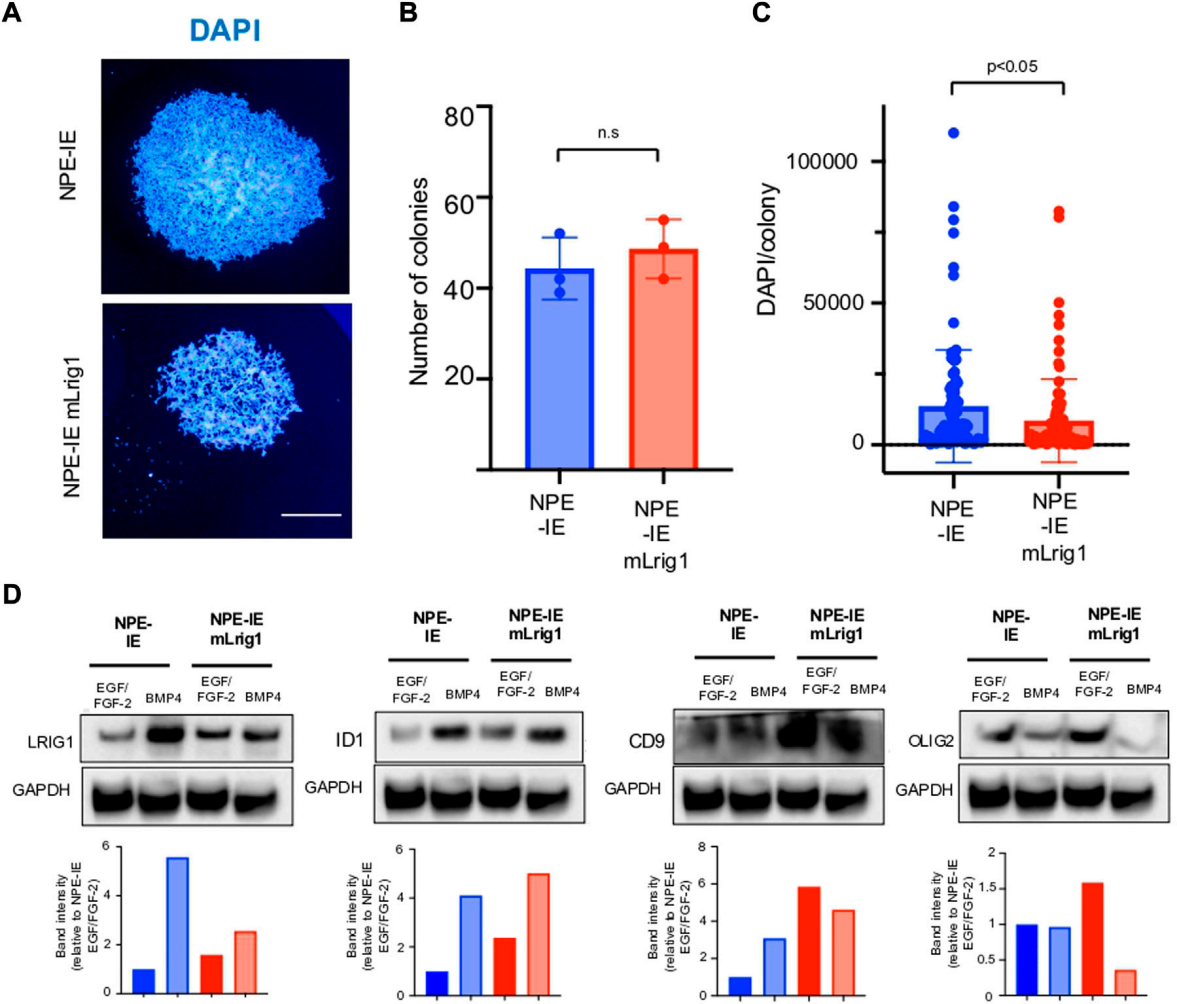
Overexpression of Lrig1 reduces GSC proliferation. **(A)** Representative fluorescence imaging of DAPI stained colonies using Operetta high-content analysis system for NPE-IE control and NPE-IE mLrig1 overexpressing cells. **(B)** Quantification of the number of colonies formed in single cell colony forming assays by NPE-IE control and NPE-IE mLrig1 overexpressing cells (n = 3) **(C)** Quantification of the size of colonies formed in single cell colony forming assays by NPE-IE control and NPE-IE mLrig1 overexpressing cells (n = 3) *p* = 0.0456. Each dot represents a single colony. **(D)** Immunoblot and quantification of LRIG1, OLIG2, ID1, CD9 and GAPDH (loading control) in NPE-IE control and NPE-IE mLrig1 overexpressing GSCs treated with EGF/FGF or BMP4 for 3 days. Quantification shown relative to GAPDH and NPE-IE EGF/FGF-2 control. Scale bar in **(A)** is 500 μm.

Using NPE-IE cells we explored the consequences of Lrig1 overexpression, to assess if this would be sufficient to suppress proliferation. NPE-IE cells were transfected with a mouse Lrig1 overexpression construct using the PiggyBac transposase system (FLAG-tagged mouse Lrig1 expressed from the CMV promoter, as in Figures 2F–H). Cells with the highest Lrig1 levels were selected by antibody-mediated FACS and expanded (Supplementary Figure S6). Single cell colony forming assays in NPE-IE mLrig1 and control NPE-IE cells revealed that while Lrig1 overexpression does not alter colony-forming capacity, the average colony size is decreased (Figures 4A–C). As expected, immunoblotting analysis found BMP treatment enhances Lrig1 expression in NPE-IE cells, consistent with elevated levels of quiescence markers Id1 and Cd9 (Figure 4D). Additionally, under proliferative conditions (EGF/FGF) NPE-IE mLrig1 cells show higher Id1 and Cd9 levels compared to control NPE-IE in EGF/FGF, supporting evidence that Lrig1 overexpression enhances quiescence *in vitro*.

### Lrig1 overexpression reduces the tumour-initiating capacity of glioblastoma stem cells *in vivo*

**FIGURE 5:**
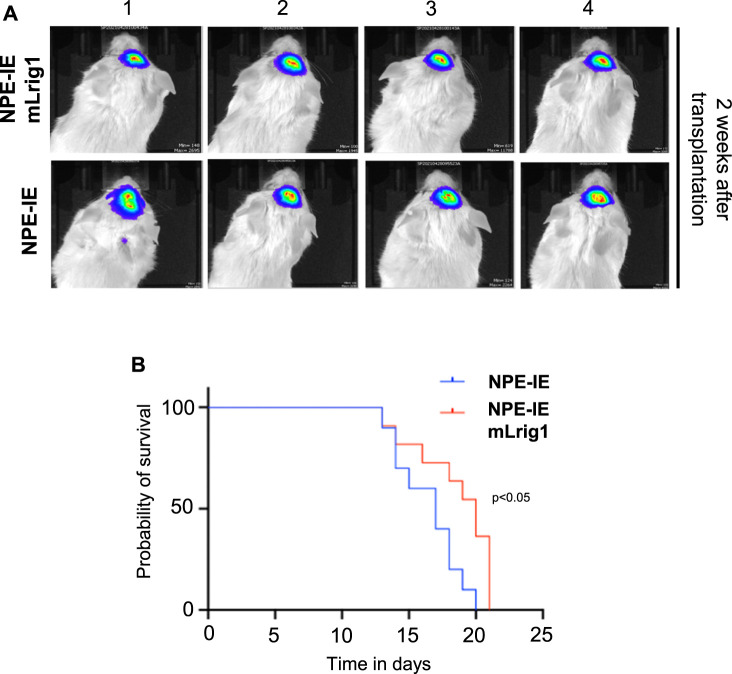
Lrig1 overexpression reduces tumour progression of GSCs. **(A)** Bioluminescent IVIS imaging of NSG mice 2 weeks following intracranial transplantation of NPE-IE and NPE-IE mLrig1 GSCs. Each image shows a different mouse (n = 4 shown for each cell type). **(B)** Kaplan Meier survival curve for mice with intracranial transplantation NPE-IE and NPE-IE mLrig1 GSCs (10–11 mice respectively). Log-rank (Mantel-Cox) test *p* = 0.0226.

To explore the effect of Lrig1 overexpression on the tumour-initiating capacity of aggressive NPE-IE GSCs, we transplanted NPE-IE mLrig1 and parallel NPE-IE controls into the striatum of NSG mice (Figure 5). Tumour progression could be tracked *in vivo* with bioluminescent (IVIS) imaging due to a luciferase reporter in NPE-IE cells. Two weeks after transplantation, IVIS imaging of luciferase signal revealed tumour formation in both mice transplanted with NPE-IE GSCs or NPE-IE mLrig1 GSCs (Figure 5A). Survival analysis over a 20-day period confirmed that mice transplanted with NPE-IE mLrig1 GSCs showed a delayed onset of symptoms and thus increased survival compared to NPE-IE control GSCs (Figure 5B). This suggests that Lrig1 overexpression reduces tumour progression *in vivo*.

Altogether, our investigations suggest that the cell surface protein Lrig1 is an important regulator of not only EGFR signalling, but also BMP responsiveness. This has important implications for our understanding of the proportions of quiescent and proliferative subpopulations in GBMs and suggests Lrig1 agonists may have value in suppressing the proliferative GSC state.

## Discussion

The survival of residual quiescent GSCs underpins GBM tumour recurrence and patient relapse following current treatments. Many recent studies suggest that quiescence is not a passive state, but rather a reversible G_0_ phase phenotype requiring active maintenance and regulation. Quiescent cells may be activated to re-enter the cell cycle and transition to a proliferative state (Cheung and Rando, 2013). It is therefore vital that we understand the molecular processes that control the maintenance of and exit from GSC quiescence, as this may enable rational design of future GBM therapies that suppress regrowth of the tumour.

It is well-known that bone morphogenetic protein (BMP) signalling induces cell-cycle exit and quiescence of normal NSCs, both *in vitro* and *in vivo* (Bonaguidi et al., 2005; Mira et al., 2010; Marqués-Torrejón et al., 2021). BMPs belong to the transforming growth factor-β (TGF-β) superfamily of cytokines that activate the BMP signalling pathway through binding to serine-threonine kinase receptors. Several studies have shown that BMPs are cytostatic in many patient-derived GSCs (i.e., suppress the tumorigenic capacity of GSCs by inducing features of astrocytic differentiation or quiescence, both *in vitro* and *in vivo*) (Nakano et al., 2008; Chirasani et al., 2010; Klose et al., 2011; Reguera-Nuñez et al., 2014; Sachdeva et al., 2019). Whether the astrocyte morphology and markers induced by BMP indicate terminal astrocytic differentiation, or rather a transition into a dormant/quiescent state remains difficult to determine without definitive markers. However, BMP pathway activation has been shown to confer resistance to conventional GBM therapies through mediating quiescence (Sachdeva et al., 2019). Among all BMP ligands tested, BMP4 elicited the strongest cytostatic effect in GSCs (Piccirillo et al., 2006). BMP-driven differentiation therapy has consequently been proposed as a potential therapeutic strategy to target GSCs (Kim and Choe, 2011). At least *in vitro* however, GSCs fail to undergo terminal differentiation and cells remain vulnerable to reversion to the GSC state upon re-exposure to mitogens (Carén et al., 2015). GSCs also use strategies to limit BMP responses, such as silencing of the BMP receptor (Lee et al., 2008).

Lrig1 has prognostic value in several cancers, including GBM, through its role as a tumour suppressor regulating EGFR activity (Lindquist et al., 2014; Yu et al., 2018; Ji et al., 2022). In this study, we uncover further evidence of a regulatory relationship between the tumour suppressor and quiescence marker Lrig1 and the BMP signalling pathway. Using both gain- and loss-of-function studies we have demonstrated that Lrig1 over-expression increases quiescence features of mouse GSCs and reduces tumorigenicity, whereas Lrig1 loss reduces quiescence and increases tumorigenicity of GSCs. These data are consistent with previous studies, where ectopic expression of Lrig1 opposed EGFRvIII driven proliferation, survival and invasion in GBM cells (Stutz et al., 2008), and RNAi of *Lrig1* promoted aggressiveness *via* EGFR/Akt/c-myc activation (Xie et al., 2013). Importantly, we show that Lrig1 KO GSCs display impaired BMP signalling, rescued by Lrig1 re-expression, while Lrig1 overexpressing cells show increased quiescence even under proliferative growth conditions. These data indicate Lrig1 regulates quiescence of GSCs via the BMP signalling pathway, and not merely the antagonism of EGFR, as has been previously reported. These dual roles may explain its potency in enabling increased proliferation of GSCs when lost. Coinciding with our work, it has been hypothesised that Lrig proteins regulate stem cell quiescence by promoting BMP signalling (Herdenberg and Hedman., 2022); our data indeed support this hypothesis for Lrig1 in normal NSCs and GSCs.

Our findings provide another explanation for the highly heterogeneous responses of GSCs to BMP treatment and we speculate that variations in Lrig1 protein levels between cells may alter BMP responsiveness (Marqués-Torrejón et al., 2021). Interestingly, Lrig1 has been found to enhance sensitivity to cytotoxic drugs, reverse multidrug resistance, and enhance radiosensitivity of serum-cultured U251 GBM cells *via* EGFR repression (Wang et al., 2012; Liu et al., 2015; Yang et al., 2015). In contrast, our results show Lrig1 KO GSCs to be more proliferative and therefore likely more sensitive to anti-mitotic therapies. Lrig1 may therefore be a useful cell surface marker to track quiescent and proliferative tumour compartments and their signalling responsiveness.

In conclusion, Lrig1 is an important regulator of the balance between proliferation and quiescence in mouse GSCs. Alongside its known roles in suppressing EGFR signals, Lrig1 operates to provide competence to respond efficiently to BMP signalling, an important inducer of quiescence. Understanding the mechanisms behind GSC quiescence and therapeutic resistance will aid future rational therapies to improve GBM patient outcomes.

## Material and methods

### Cell culture

NSCs and GSCs were maintained *in vitro* in serum-free basal medium supplemented with N2 and B27 (Life Technologies), Laminin-1 (Sigma, 1 μg/ml) and growth factors EGF and FGF-2 (Peprotech # 100–18b and #315–09, 10 ng/ml each) (Pollard et al., 2009). For single cell colony-forming assays NSCs were plated as single cells in 96 multi-well plates. Treatments with BMP-4 were for 3 days (5,10 and 20 ng/ml, Peprotech, AF-120–05 ET-100).

### Animals

Mice were maintained on a regular diet in a pathogen-free facility on a 12-h light/dark cycle with unlimited access to food and water. Intracranial transplantation of GSCs was performed using a stereotaxic frame to inject 2,00,000 cells resuspended in 2 μL of NSC media into the striatum of 6- to 8-week-old male NOD/SCID/GAMMA (NSG) mice, following administration of isoflourane general anesthesia and Rimadyl analgesic. Coordinates were 0.6 mm anterior and 1.5 mm lateral to the bregma and 2.4 mm deep. Cell preparation and procedures were performed as previously described (Pollard et al., 2009). Bioluminescent imaging was performed 20 min after D-Luciferin (50 mg/kg) subcutaneous injection, using the IVIS Lumina LT Series III instrument (Perkin Elmer). Animals were fixed in a stereotactic apparatus Stoelting (SKU S51725) and an automatic injector KD Scientific product, Model KDS-311-CE, catalog number 78-9311UU. Animals were anaesthetised and transcardially perfused with 4% paraformaldehyde in 0.1 M PBS and brains processed for vibratome sectioning (Leica VT1,000S vibratome). For *in vivo* detection of slowly proliferating cells, CldU (invitrogen) was dissolved in PBS at 5 mg/ml and injected (IP) at 1 mg/20 g mouse, 200 ul once daily for 3 days. After 3 weeks, mice were sacrificed and CldU was assessed by IHC. All animal procedures were performed under United Kingdom Government Home Office approved Procedure Project License (PC0395462) and following approval of the local Animal Welfare and Ethical Review Body (AWERB).

### Organotypic co-culture

For organotypic co-culture, young adult (5 weeks old) C57BL/6 SCRM (BL6) mice were used. They were sacrificed by cervical dislocation and processed as previously described in (Marques-Torrejon et al., 2018). Samples were blocked in 10% normal goat serum and 0.2% Triton X-100 in PBS for 1 h and incubated for 48 h in blocking buffer with the appropriate primary antibodies: GFAP (1:500, Z0334 DAKO) KI67 (1:100 Thermo RM9106), SOX2 (1:100, AB5603, Millipore), NESTIN (1:10, Rat 401, Developmental Studies Hybridoma Bank), p53 (1:500, ab6326, Cell signalling), LRIG1 (1:100, R&D, AF3688), CD9 (1:100, 14–0091–82, eBioscience, 1:500), RFP (1:500, Abcam 62,341), BrdU (1; 1,000 Abcam, ab6326), SSEA1 (1:500, Biolegend, ab63260), Vimentin-40E (1:50, DSHB), CD133 (1:500, Milllipore, ab6326), CD9 (1; 500,14–0091–82, eBioscience), and pSMAD1/5 (1:500, Cell signalling, 9,516).

### Immunocytochemistry

Cells were fixed with 4% paraformaldehyde for 20 min, incubated in blocking buffer (10% normal goat serum and 0.2% Triton X-100 in 0.1 M phosphate buffer saline) for 30 min, and incubated overnight at 4 C with the indicated primary antibodies. After several washes with PBS, immunoreactivity was detected using appropriate Alexa Fluor-conjugated (Life Technologies) secondary antibodies (1:500) diluted in blocking buffer. Cells were counterstained with 4′,6′, -diamidino-2-phenylindole (DAPI) or DRAQ5 and mounted with Fluorsave (Calbiochem). Manufacturer’s instructions were followed for both EdU [Click it Thermo Fisher (C10337)] and for protein synthesis [Click it Thermo Fisher OP-Puro (C10456)] assays. Images were taken and analyzed using Confocal (Leica SP8, four and five detectors) and Nikon TiE microscopes. CellTrace™ quantification was measured using FIJI software (Fiji version:2.0.0-rc-69). A negative control with no fluorescence was used.

### Flow cytometry and sorting

For cell sorting using antibodies, cells were split and washed with PBS. The cell pellet was incubated with the primary antibody LRIG1 (1:200, R&D AF3688) diluted in PBS with 4% FCS at 37 C for 1 h. After washing with PBS, the pellet was incubated in secondary Alexa Fluor-conjugated (Life Technologies) antibodies diluted (1:500) in PBS-4%FCS for 10 min. BD LSR Fortessa (SORP) and BD Fusion Cell Sorters were used for analysis and sorting, respectively.

### Western immunoblotting

Immunoblotting was performed using standard protocols. Antibodies were diluted in 5% milk powder in PBS Triton 0.1%, and protein detection was carried out with HRP-coupled secondary antibodies and X-ray films. The following primary antibodies were used: LRIG1 (1:100, R&D, AF3688), EGFR (1:1,000, D38B1, Cell Signaling, #4267), EGFR-p (1:1,000, Tyr 1,068, Cell Signaling, #3,777), pSMAD1-5 (1:1,000, Cell Signaling, #9,516), SMAD1 (1:1,000, Cell Signaling, #9,743), ppERK1/2 (1:1,000, Cell Signaling #9101), ERK1/2 (1:1,000, Cell Signaling 4,695), Olig2 (1:3,000, Millipore, #9610), Id1 (1:2,500; Biocheck # BCH-1/195–14), CD9 (1:100; Millopore, #CBL 162), BLBP (Abcam, 1: 1,000; #3,24,230, GAPDH (1:50,000; GenTex, GTX627408). No accutase was used when collecting cell pellets. Western blot quantification was performed in ImageJ. Each band was selected, and the mean intensity measured in arbitrary units. The intensity of each band was then divided by the intensity of the respective GAPDH band to normalise the loading. Normalised values were then divided by the normalised intensity of the control band to give the fold change relative to the control for each blot.

### Transfection and derivation of clonal lines

Synthetic Alt-R crRNA and tracrRNA were manufactured by Integrated DNA Technologies. dsDNA block oligonucleotides were manufactured by Twist Bioscience and recombinant Cas9 protein was made in-house. NSCs were transfected using PiggyBac-GFP-Luc BSD plasmid with EGFRvIII and the guides for p53 (Gangoso et al., 2021). For *Lrig1* mutant cells, design and construction of CRISPR sgRNAs are described in (Bressan et al., 2017). We disrupted Exon1 based on (Suzuki et al., 2002). We used the 4D Amaxa nucleofector and DN-100 program. For *Lrig1* deletion we used two RNA guides with the following sequences: AAG​GCG​ACT​CTC​AGC​GCG​GC and TAC​TCA​CAG​GCT​GCG​CGT​CC (Marqués-Torrejón et al., 2021). To overexpress *Lrig1*, we used the m*Lrig1*-CFLAG plasmid (mouse LRIG1 expressed from CMV promoter; gift from Prof Kim Jensen, University of Copenhagen).

### PCR-based p53 genotyping cells

For genomic DNA isolation, each well of a confluent 24-well plate was lysed with 40 µL of lysis buffer (0.45% NP40, 0.45% Tween20, 1x NEB LongAmp PCR buffer) containing 0.2 mg ml−1 proteinase K (Sigma). After a 2 h digestion at 55 C, samples were heated to 95 C (10 min) and 1–2 µL of the lysate was used in a 10 µL PCR reaction. PCR mix consisted of 0.2 µL DMSO (100% v/v, Sigma), 0.3 µL dNTPs (10 mM, Thermo Fisher Scientific), 2.0 µL 5x LongAMP buffer (NEB), 0.4 µL LongAMP Taq DNA polymerase (NEB), and 12 pmol of each primer. Thermal cycling was performed using the following conditions: one cycle 94 C for 3 min; 35 cycles (94 C for 30 s, 60 C for 30 s, 65 C for 2 min); followed by a final extension at 65 C (Gangoso et al., 2021).

### Quantitative real-time RT-PCR

RNA was extracted using the RNeasy spin column kit (Qiagen), plus DNase treatment to eliminate gDNA. cDNA was generated with SuperScript III (Invitrogen), and quantitative RT-PCR was performed using Taqman universal PCR Master Mix (Applied Biosystems). The following Taqman assays (Life Technologies) were used: Bmpr1a Mm00477650_m1, Bmpr1b Mm03023971_m1, Bmpr2 Mm00432134_m1, Id1 Mm00775963_g1, Gapdh Mm00775963_g1.

### Statistical methods

Results are presented as mean ± SD of a number (*n*) of independent experiments. Statistical significance was determined by two-tailed Student’s t-tests using GraphPad (version 9.0.0). Treatment experiments were analysed using paired *t*-tests. Significance of Kaplan Meier survival curves determined by Log-rank (Mantel-Cox) test. When comparisons were performed with relative values (normalised values and percentages), data were normalised by using an arc-sen transformation. Values of *p* < 0.05 were considered statistically significant. Box and whisker plots show the mean, and maximum and minimum values.

## Data Availability

The raw data supporting the conclusion of this article will be made available by the authors, without undue reservation.

## Supplementary material

SUPPLEMENTARY FIGURE S1
**(A)** Schematic describing the procedure of transforming mouse NSCs into GSCs through p53 deletion and EGFRvIII overexpression. Clone 6 was used in the experiments. **(B)** PCR genotyping for p53 deletion in clonal cell lines 4–12. Untransfected parental shows the wild-type PCR band size. **(C)** Immunostaining for p53 (red) and nuclear counterstaining with DAPI (blue) in cells treated and not treated with Adriamycin, where Adriamycin rises p53 levels dramatically after DNA damage. **(D)** Immunoblot for EGFR Y1068 in clonal cell lines 4, 6, 10 and 12. GAPDH is used as a housekeeping loading control. Scale bar in **(C)** is 50 μm.

SUPPLEMENTARY FIGURE S2
**(A)** Schematic describing the procedure for orthotopic transplantation of Fucci PE GSCs into NSG mice. **(B)** Representative live imaging of the Fucci reporters (mCherry-Cdt1 and aVenus-hGem) in the tumours dissected from mice treated with saline or TMZ. TMZ abolishes Venus signal (cells in S/G2/M phase) (n = 4 per condition). **(C)** Immunostaining for KI67 (green), CldU (yellow), Fucci mCherry-Cdt1 reporter (red) and nuclear counterstaining with DAPI (blue), in the tumours dissected from mice treated with saline or TMZ. **(D)** Quantification of percentage of Ki67 and CldU positive cells (P = 0.0446 and 0.031 respectively). Scale bar in **(C)** is 100 μm.

SUPPLEMENTARY FIGURE S3
**(A)** Schematic describing the strategy for *Lrig1* deletion from mouse GSCs. **(B)** Flow cytometry plots showing antibody-based selection of the Lrig1 negative population following transfection. Plot i) shows the negative control with no antibody. Plot ii) shows the parental full stained control with no negative population. Plot iii) shows the transfected population with both Lrig1 KO and Lrig1-high expressing cells present. **(C)** Immunoblot for LRIG1, P-EGFR and GAPDH (housekeeping loading control) in GSCs with WT Lrig1 (untransfected parental) and Lrig1 KO.

SUPPLEMENTARY FIGURE S4
*Bmpr1a, Bmpr1b, Bmpr2* and *Id1* expression measured by qRT-PCR in the Fucci PE Lrig1 KO GSCs (relative to expression in Fucci PE Lrig1 WT GSCs which equals 1), n = 3, mean ± SD.

SUPPLEMENTARY FIGURE S5 Immunostaining and quantification for CD9, NESTIN, GFAP, SOX2, KI67, VIMENTIN, SSEA1, CD133 (red) and nuclear counterstaining with DAPI (grey) in the tumours from mice transplanted with Lrig1 WT or Lrig1 KO GSCs (PE) (n = 4). P = 0.0243 and p = 0.0380 for GFAP and Ki67, respectively. Scale bar is 50 μm.

SUPPLEMENTARY FIGURE S6 Flow cytometry plots showing strategy to select NPE-IE cells with the highest Lrig1 levels by antibody-mediated FACS. Lrig1 antibody was used to select the highest levels of Lrig1. V450-50-A is DAPI to select the live population. B530/30-A represents the GFP reporter in all cells and YG670/14-A represents Lrig1. The P4 population expressed the highest levels of Lrig1; this population was collected and expanded, with the resulting population termed NPE-IE mLrig1.

## References

[B1] Arbab A. S. Rashid M. H. Angara K. Borin T. F. Lin P. C. Jain M. (2017). Major challenges and potential microenvironment-targeted therapies in glioblastoma. Int. J. Mol. Sci. 18, E2732. 10.3390/ijms18122732 29258180 PMC5751333

[B2] Bao Z. Zhang C. Yan W. Liu Y. Li M. Zhang W. (2013). BMP4, a strong better prognosis predictor, has a subtype preference and cell development association in gliomas. J. Transl. Med. 11, 100–107. 10.1186/1479-5876-11-100 23590708 PMC3637580

[B3] Batlle E. Clevers H. (2017). Cancer stem cells revisited. Nat. Med. 23, 1124–1134. 10.1038/nm.4409 28985214

[B4] Beier D. Hau P. Proescholdt M. Lohmeier A. Wischhusen J. Oefner P. J. (2007). CD133+ and CD133- glioblastoma-derived cancer stem cells show differential growth characteristics and molecular profiles. Cancer Res. 67, 4010–4015. 10.1158/0008-5472.CAN-06-4180 17483311

[B5] Bonaguidi M. A. Mcguire T. Hu M. Kan L. Samanta J. Kessler J. A. (2005). LIF and BMP signaling generate separate and discrete types of GFAP-expressing cells. Development 132, 5503–5514. 10.1242/dev.02166 16314487

[B6] Bressan R. B. Dewari P. S. Kalantzaki M. Gangoso E. Matjusaitis M. Garcia-Diaz C. (2017). Efficient CRISPR/Cas9-assisted gene targeting enables rapid and precise genetic manipulation of mammalian neural stem cells. Development 144, 635–648. 10.1242/dev.140855 28096221 PMC5312033

[B7] Carén H. Stricker S. H. Bulstrode H. Gagrica S. Johnstone E. Bartlett T. E. (2015). Glioblastoma stem cells respond to differentiation cues but fail to undergo commitment and terminal cell-cycle arrest. Stem Cell Rep. 5, 829–842. 10.1016/j.stemcr.2015.09.014 PMC4649264 26607953

[B8] Chen J. Li Y. Yu T. S. McKay R. M. Burns D. K. Kernie S. G. (2012). A restricted cell population propagates glioblastoma growth after chemotherapy. Nature 488, 522–526. 10.1038/nature11287 22854781 PMC3427400

[B9] Cheung T. H. Rando T. A. (2013). Molecular regulation of stem cell quiescence. Nat. Rev. Mol. Cell Biol. 14, 329–340. 10.1038/nrm3591 23698583 PMC3808888

[B10] Chirasani S. R. Sternjak A. Wend P. Momma S. Campos B. Herrmann I. M. (2010). Bone morphogenetic protein-7 release from endogenous neural precursor cells suppresses the tumourigenicity of stem-like glioblastoma cells. Brain 133, 1961–1972. 10.1093/brain/awq128 20513660

[B11] Deleyrolle L. P. Harding A. Cato K. Siebzehnrubl F. A. Rahman M. Azari H. (2011). Evidence for label-retaining tumour-initiating cells in human glioblastoma. Brain 134, 1331–1343. 10.1093/brain/awr081 21515906 PMC3097894

[B12] Fernandes C. Costa A. Osório L. Lago R. C. Linhares P. Carvalho B. (2017). in Current standards of care in glioblastoma TherapyGlioblastoma. Editor De Vleeschouwer S. (Brisbane AU. Codon Publications). 29251860

[B13] Galli R. Binda E. Orfanelli U. Cipelletti B. Gritti A. De Vitis S. (2004). Isolation and characterization of tumorigenic, stem-like neural precursors from human glioblastoma. Cancer Res. 64, 7011–7021. 10.1158/0008-5472.can-04-1364 15466194

[B14] Gangoso E. Southgate B. Bradley L. Rus S. Galvez-Cancino F. McGivern N. (2021). Glioblastomas acquire myeloid-affiliated transcriptional programs via epigenetic immunoediting to elicit immune evasion. Cell 184, 2454–2470.e26. e26. 10.1016/j.cell.2021.03.023 33857425 PMC8099351

[B15] Gross R. E. Mehler M. F. Mabie P. C. Zang Z. Santschi L. Kessler J. A. (1996). Bone morphogenetic proteins promote astroglial lineage commitment by mammalian subventricular zone progenitor cells. Neuron 17, 595–606. 10.1016/S0896-6273(00)80193-2 8893018

[B16] Gur G. Rubin C. Katz M. Amit I. Citri A. Nilsson J. (2004). LRIG1 restricts growth factor signaling by enhancing receptor ubiquitylation and degradation. EMBO J. 23, 3270–3281. 10.1038/sj.emboj.7600342 15282549 PMC514515

[B17] Haas T. L. Sciuto M. R. Brunetto L. Valvo C. Signore M. Fiori M. E. (2017). Integrin α7 is a functional marker and potential therapeutic target in glioblastoma. Cell Stem Cell 21, 35–50. e9. 10.1016/j.stem.2017.04.009 28602620

[B18] Hedman H. Henriksson R. (2007). LRIG inhibitors of growth factor signalling - double-edged swords in human cancer? Eur. J. Cancer 43, 676–682. 10.1016/j.ejca.2006.10.021 17239582

[B19] Herdenberg C. Hedman H. (2022). Hypothesis: Do LRIG proteins regulate stem cell quiescence by promoting BMP signaling? Stem Cell Rev. Rep. 10.1007/s12015-022-10442-9 PMC9823064 35969315

[B20] Herdenberg C. Mutie P. M. Billing O. Abdullah A. Strawbridge R. J. Dahlman I. (2021). LRIG proteins regulate lipid metabolism via BMP signaling and affect the risk of type 2 diabetes. Commun. Biol. 4, 90–15. 10.1038/s42003-020-01613-w 33469151 PMC7815736

[B21] Ji Y. Kumar R. Gokhale A. Chao H. P. Rycaj K. Chen X. (2022). LRIG1, a regulator of stem cell quiescence and a pleiotropic feedback tumor suppressor. Semin. Cancer Biol. 82, 120–133. 10.1016/j.semcancer.2020.12.016 33476721 PMC8286266

[B22] Johansson M. Oudin A. Tiemann K. Bernard A. Golebiewska A. Keunen O. (2013). The soluble form of the tumor suppressor Lrig1 potently inhibits *in vivo* glioma growth irrespective of EGF receptor status. Neuro. Oncol. 15, 1200–1211. 10.1093/neuonc/not054 23723255 PMC3748912

[B23] Khan I. Mahfooz S. Elbasan E. B. Karacam B. Oztanir M. N. Hatiboglu M. A. (2021). Targeting glioblastoma: The current state of different therapeutic approaches. Curr. Neuropharmacol. 19, 1701–1715. 10.2174/1570159X19666210113152108 33441071 PMC8977637

[B24] Kim M. Choe S. (2011). BMPs and their clinical potentials. BMB Rep. 44, 619–634. 10.5483/BMBRep.2011.44.10.619 22026995 PMC3423198

[B25] Klose A. Waerzeggers Y. Monfared P. Vukicevic S. Kaijzel E. L. Winkeler A. (2011). Imaging bone morphogenetic protein 7 induced cell cycle arrest in experimental gliomas. Neoplasia 13, 276–285. 10.1593/neo.101540 21390190 PMC3050870

[B26] Kreso A. Dick J. E. (2014). Evolution of the cancer stem cell model. Cell Stem Cell 14, 275–291. 10.1016/j.stem.2014.02.006 24607403

[B27] Laederich M. B. Funes-Duran M. Yen L. Ingalla E. Wu X. Carraway K. L. 3rd (2004). The leucine-rich repeat protein LRIG1 is a negative regulator of ErbB family receptor tyrosine kinases. J. Biol. Chem. 279, 47050–47056. 10.1074/jbc.M409703200 15345710

[B28] Lathia J. D. Gallagher J. Heddleston J. M. Wang J. Eyler C. E. MacSwords J. (2010). Integrin Alpha 6 regulates glioblastoma stem cells. Cell Stem Cell 6, 421–432. 10.1016/j.stem.2010.02.018 20452317 PMC2884275

[B29] Lee J. Son M. J. Woolard K. Donin N. M. Li A. Cheng C. H. (2008). Epigenetic-mediated dysfunction of the bone morphogenetic protein pathway inhibits differentiation of glioblastoma-initiating cells. Cancer Cell 13, 69–80. 10.1016/j.ccr.2007.12.005 18167341 PMC2835498

[B30] Lindquist D. Kvarnbrink S. Henriksson R. Hedman H. (2014). LRIG and cancer prognosis. Acta Oncol. 53, 1135–1142. 10.3109/0284186X.2014.953258 25180912 PMC4438349

[B31] Liu B. Guo Z. Dong H. Daofeng T. Cai Q. Ji B. (2015). LRIG1, human EGFR inhibitor, reverses multidrug resistance through modulation of ABCB1 and ABCG2. Brain Res. 1611, 93–100. 10.1016/j.brainres.2015.03.023 25801120

[B32] Mao F. Wang B. Xiao Q. Cheng F. Lei T. Guo D. (2017). LRIG proteins in glioma: Functional roles, molecular mechanisms, and potential clinical implications. J. Neurol. Sci. 383, 56–60. 10.1016/j.jns.2017.10.025 29246624

[B33] Marques-Torrejon M. A. Gangoso E. Pollard S. M. (2018). Modelling glioblastoma tumour-host cell interactions using adult brain organotypic slice co-culture. Dis. Model. Mech. 11, dmm031435. 10.1242/dmm.031435 29196443 PMC5894940

[B34] Marqués-Torrejón M. Á. Williams C. A. C. Southgate B. Alfazema N. Clements M. P. Garcia-Diaz C. (2021). LRIG1 is a gatekeeper to exit from quiescence in adult neural stem cells. Nat. Commun. 12, 2594. 10.1038/s41467-021-22813-w 33972529 PMC8110534

[B35] Melin B. S. Barnholtz-Sloan J. S. Wrensch M. R. Johansen C. Il’yasova D. Kinnersley B. (2017). Genome-wide association study of glioma subtypes identifies specific differences in genetic susceptibility to glioblastoma and non-glioblastoma tumors. Nat. Genet. 49, 789–794. 10.1038/ng.3823 28346443 PMC5558246

[B36] Mira H. Andreu Z. Suh H. Chichung Lie D. Jessberger S. Consiglio A. (2010). Signaling through BMPR-IA regulates quiescence and long-term activity of neural stem cells in the adult hippocampus. Cell Stem Cell 7, 78–89. 10.1016/j.stem.2010.04.016 20621052

[B37] Mort R. L. Ford M. J. Sakaue-Sawano A. Lindstrom N. O. Casadio A. Douglas A. T. (2014). Fucci2a: A bicistronic cell cycle reporter that allows cre mediated tissue specific expression in mice. Cell Cycle 13, 2681–2696. 10.4161/15384101.2015.945381 25486356 PMC4613862

[B38] Nakano I. Saigusa K. Kornblum H. I. (2008). BMPing off glioma stem cells. Cancer Cell 13, 3–4. 10.1016/j.ccr.2007.12.018 18167333

[B39] Nassar D. Blanpain C. (2016). Cancer stem cells: Basic concepts and therapeutic implications. Annu. Rev. Pathol. 11, 47–76. 10.1146/annurev-pathol-012615-044438 27193450

[B40] Ogden A. T. Waziri A. E. Lochhead R. A. Fusco D. Lopez K. Ellis J. A. (2008). Identification of A2B5+CD133- tumor-initiating cells in adult human gliomas. Neurosurgery 62, 505–514. 10.1227/01.neu.0000316019.28421.95 18382330

[B41] Piccirillo S. G. M. Reynolds B. A. Zanetti N. Lamorte G. Binda E. Broggi G. (2006). Bone morphogenetic proteins inhibit the tumorigenic potential of human brain tumour-initiating cells. Nature 444, 761–765. 10.1038/nature05349 17151667

[B42] Pollard S. M. Yoshikawa K. Clarke I. D. Danovi D. Stricker S. Russell R. (2009). Glioma stem cell lines expanded in adherent culture have tumor-specific phenotypes and are suitable for chemical and genetic screens. Cell Stem Cell 4, 568–580. 10.1016/j.stem.2009.03.014 19497285

[B43] Powell A. E. Wang Y. Li Y. Poulin E. J. Means A. L. Washington M. K. (2012). The pan-ErbB negative regulator Lrig1 is an intestinal stem cell marker that functions as a tumor suppressor. Cell 149, 146–158. 10.1016/j.cell.2012.02.042 22464327 PMC3563328

[B44] Reguera-Nuñez E. Roca C. Hardy E. de la Fuente M. Csaba N. Garcia-Fuentes M. (2014). Implantable controlled release devices for BMP-7 delivery and suppression of glioblastoma initiating cells. Biomaterials 35, 2859–2867. 10.1016/j.biomaterials.2013.12.001 24406213

[B45] Rouam S. Moreau T. Broët P. (2010). Identifying common prognostic factors in genomic cancer studies: A novel index for censored outcomes. BMC Bioinforma. 11, 150. 10.1186/1471-2105-11-150 PMC2863163 20334636

[B46] Sachdeva R. Wu M. Johnson K. Kim H. Celebre A. Shahzad U. (2019). BMP signaling mediates glioma stem cell quiescence and confers treatment resistance in glioblastoma. Sci. Rep. 9, 14569. 10.1038/s41598-019-51270-1 31602000 PMC6787003

[B47] Singh S. K. Hawkins C. Clarke I. D. Squire J. A. Bayani J. Hide T. (2004). Identification of human brain tumour initiating cells. Nature 432, 396–401. 10.1038/nature03128 15549107

[B48] Son M. J. Woolard K. Nam D. H. Lee J. Fine H. A. (2009). SSEA-1 is an enrichment marker for tumor-initiating cells in human glioblastoma. Cell Stem Cell 4, 440–452. 10.1016/j.stem.2009.03.003 19427293 PMC7227614

[B49] Stupp R. Mason W. P. Bentvan den M. J. Weller M. Fisher B. Taphoorn M. J. (2005). Radiotherapy plus concomitant and adjuvant temozolomide for glioblastoma. N. Engl. J. Med. 352, 987–996. 10.1056/NEJMoa043330 15758009

[B50] Stutz M. A. Shattuck D. L. Laederich M. B. Carraway K. L. Sweeney C. (2008). LRIG1 negatively regulates the oncogenic EGF receptor mutant EGFRvIII. Oncogene 27, 5741–5752. 10.1038/onc.2008.185 18542056 PMC3175812

[B51] Suzuki Y. Miura H. Tanemura A. Kobayashi K. Kondoh G. Sano S. (2002). Targeted disruption of LIG-1 gene results in psoriasiform epidermal hyperplasia. FEBS Lett. 521, 67–71. 10.1016/S0014-5793(02)02824-7 12067728

[B52] Villa C. Miquel C. Mosses D. Bernier M. Di Stefano A. L. (2018). The 2016 World Health Organization classification of tumours of the central nervous system. Medicale 47, e187. 10.1016/j.lpm.2018.04.015 30449638

[B53] Wang X. Xiao Q. Xing X. Tian C. Zhang H. Ye F. (2012). LRIG1 enhances cisplatin sensitivity of glioma cell lines. Oncol. Res. 20, 205–211. 10.3727/096504013x13589503482770 23581227

[B54] Wen P. Y. Weller M. Lee E. Q. Alexander B. M. Barnholtz-Sloan J. S. Barthel F. P. (2020). Glioblastoma in adults: A society for neuro-oncology (SNO) and European society of neuro-oncology (eano) consensus review on current management and future directions. Neuro. Oncol. 22, 1073–1113. 10.1093/neuonc/noaa106 32328653 PMC7594557

[B55] Xie R. Yang H. Xiao Q. Mao F. Zhang S. Ye F. (2013). Downregulation of LRIG1 expression by RNA interference promotes the aggressive properties of glioma cells via EGFR/Akt/c-Myc activation. Oncol. Rep. 29, 177–184. 10.3892/or.2012.2102 23124613

[B56] Yang J. A. Liu B. H. Shao L. M. Guo Z. T. Yang Q. Wu L. Q. (2015). LRIG1 enhances the radiosensitivity of radioresistant human glioblastoma U251 cells via attenuation of the EGFR/Akt signaling pathway. Int. J. Clin. Exp. Pathol. 8, 3580–3590. 26097540 PMC4466927

[B57] Yu S. Yang M. Lim K. M. Cho Y. Kim H. Lee K. (2018). Expression of LRIG1, a negative regulator of EGFR, is dynamically altered during different stages of gastric carcinogenesis. Am. J. Pathol. 188, 2912–2923. 10.1016/j.ajpath.2018.08.006 30248341 PMC6334257

