# Supplementary figures and images for: Lrig1 regulates the balance between proliferation and quiescence in glioblastoma stem cells
Source: Front Cell Dev Biol. 2022 Oct 26;10:983097. doi: 10.3389/fcell.2022.983097 (PMC9677454; doi:10.3389/fcell.2022.983097)

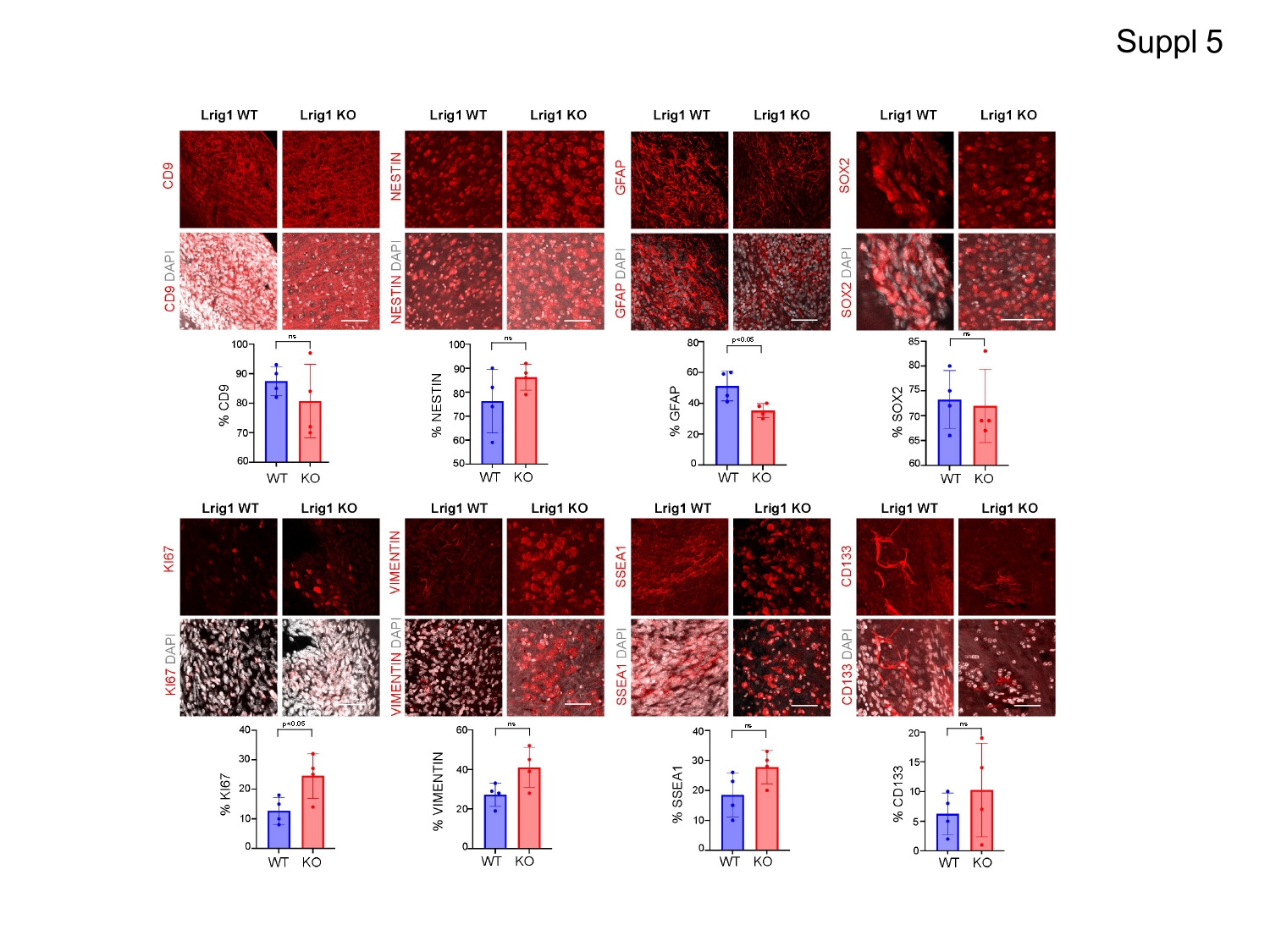

Supplement: Supplementary file 1 [file Image5.jpg]

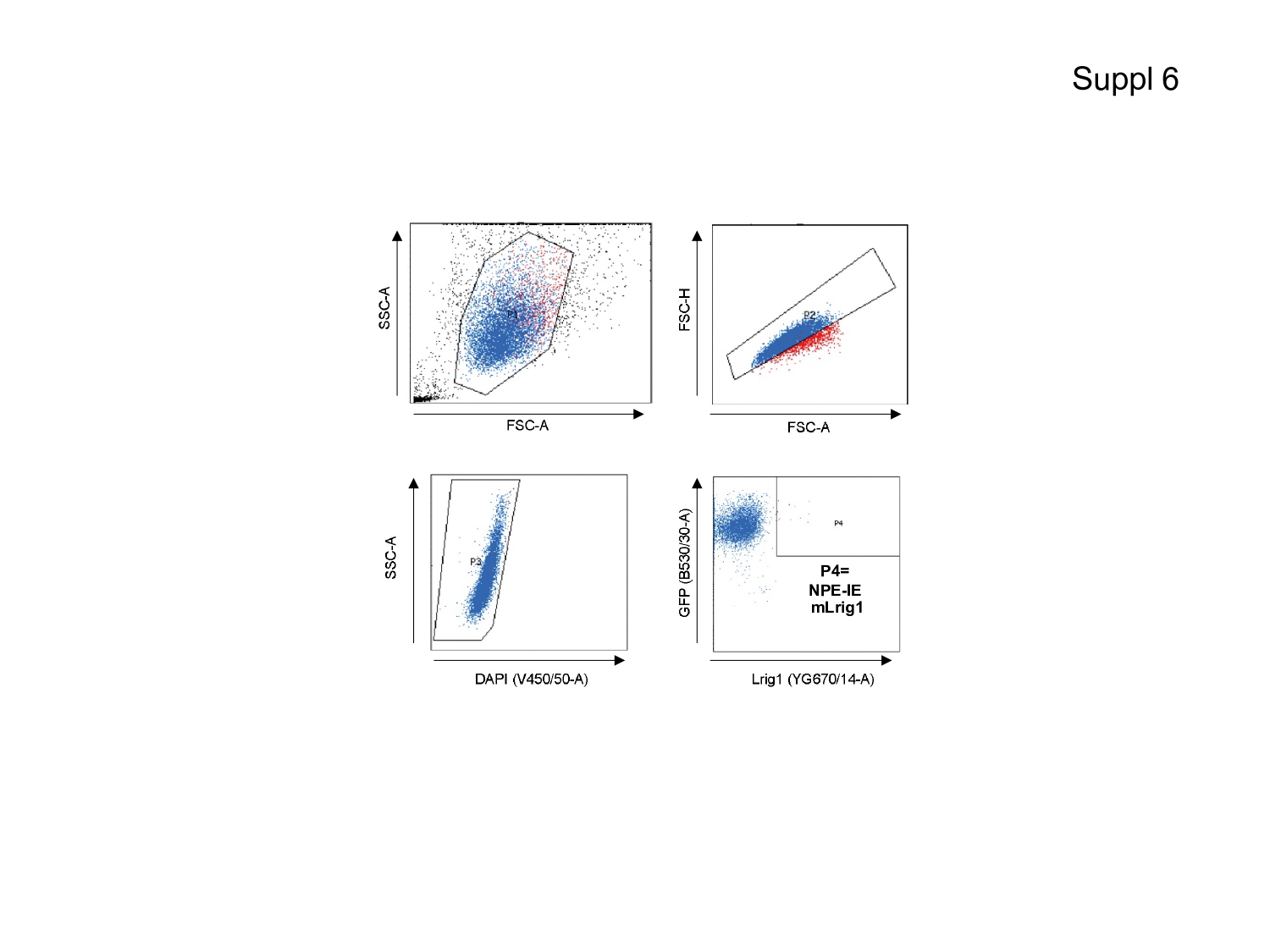

Supplement: Supplementary file 2 [file Image6.jpg]

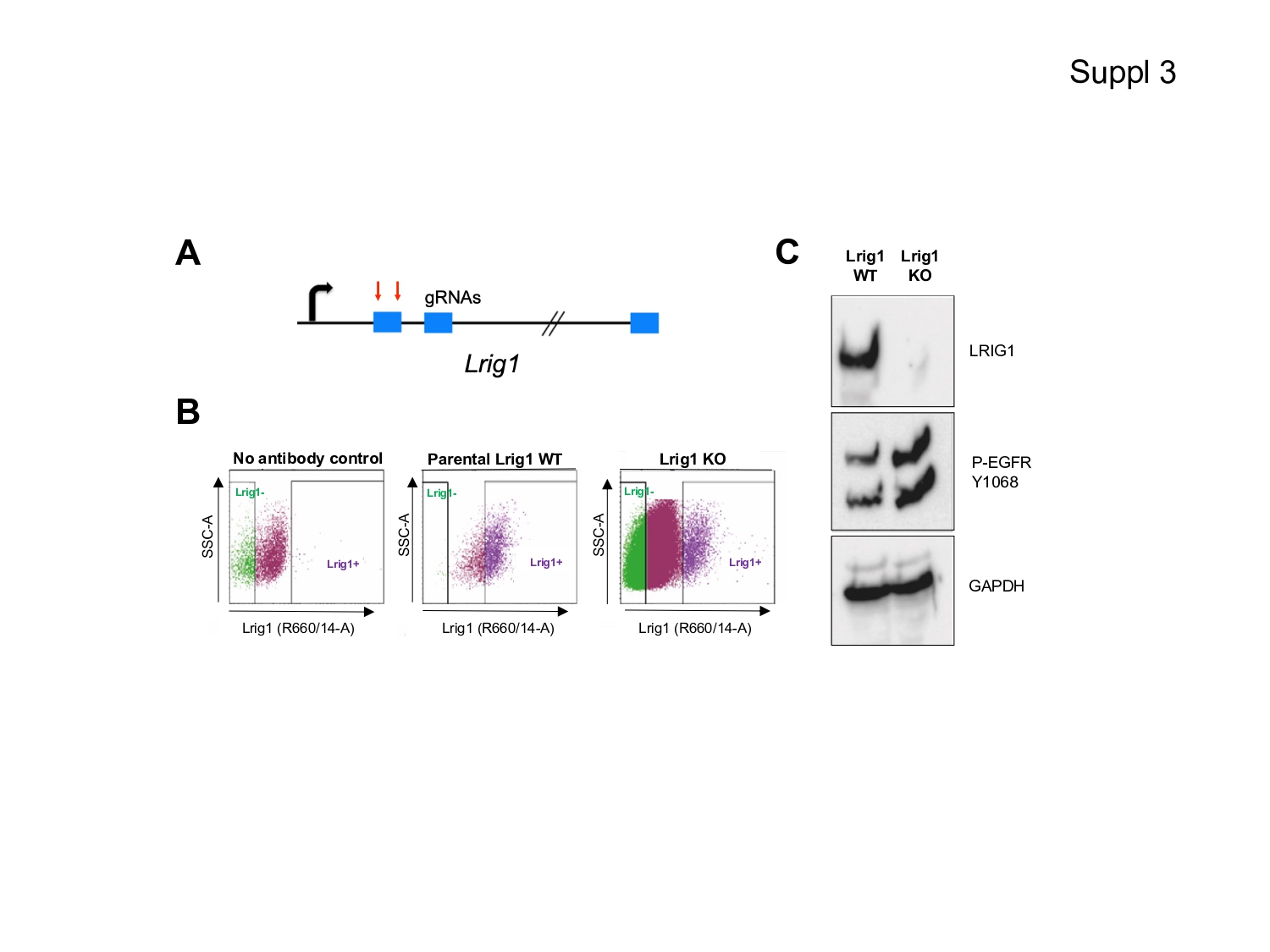

Supplement: Supplementary file 3 [file Image3.jpg]

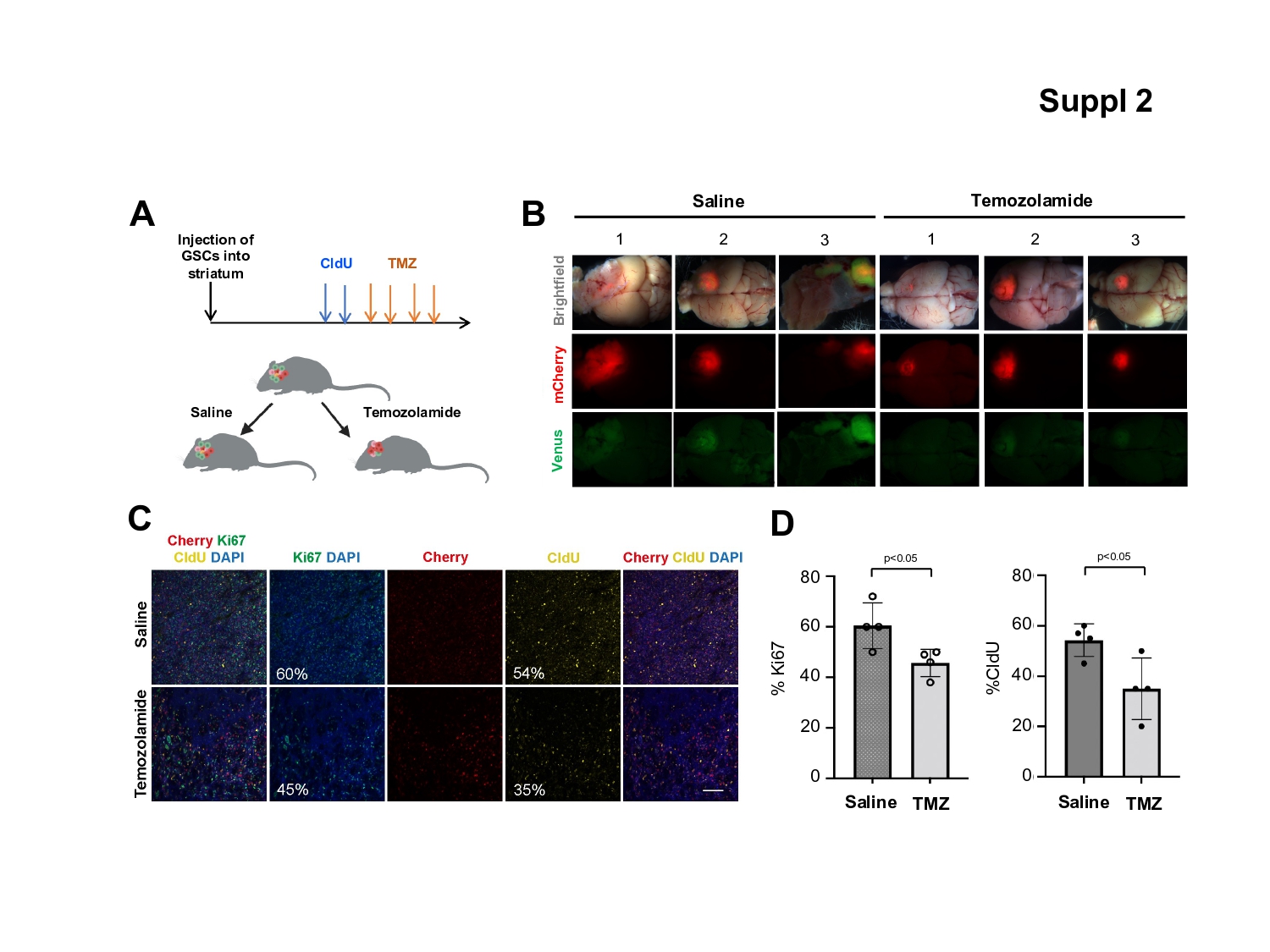

Supplement: Supplementary file 4 [file Image2.jpg]

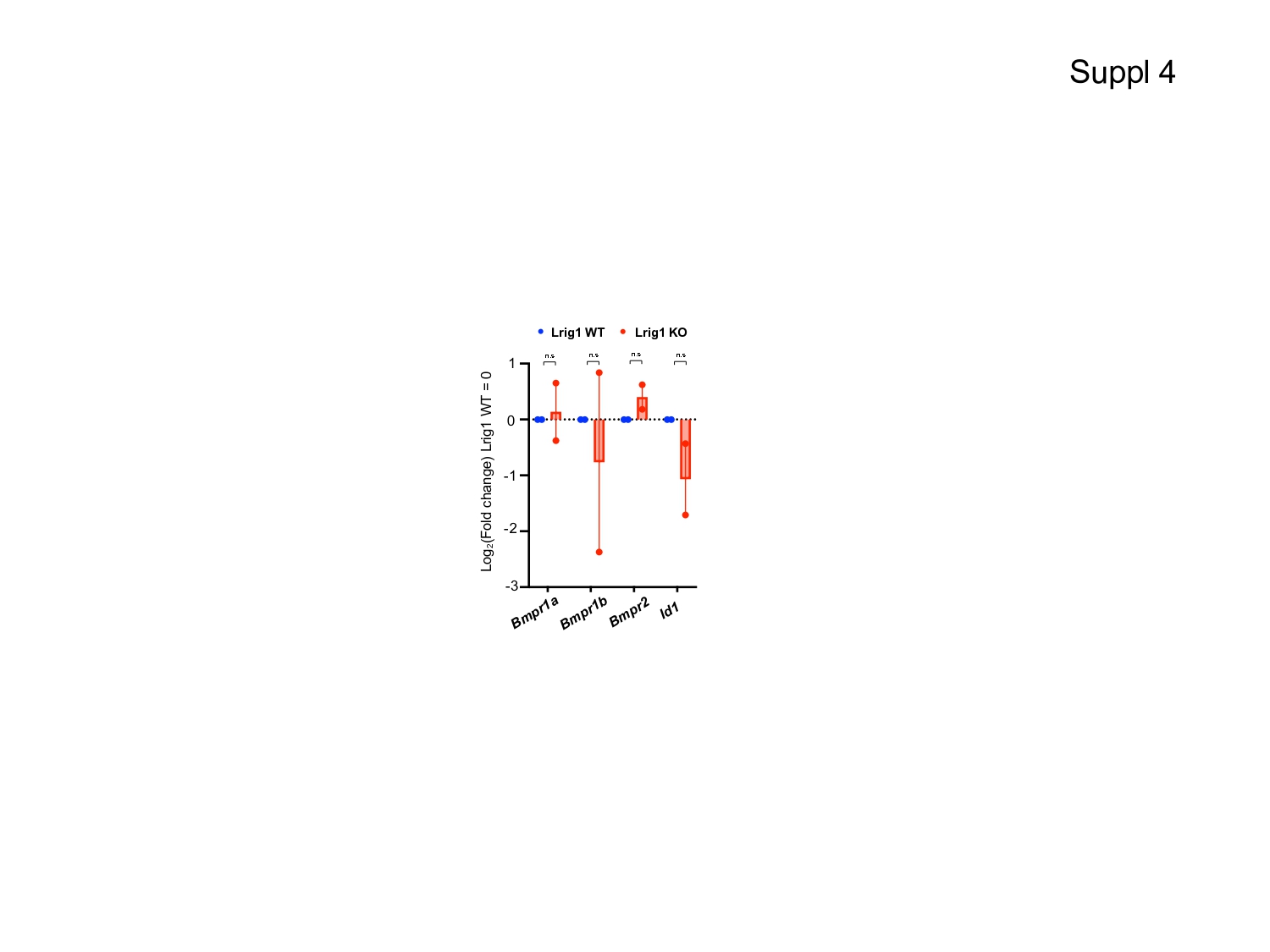

Supplement: Supplementary file 5 [file Image4.jpg]

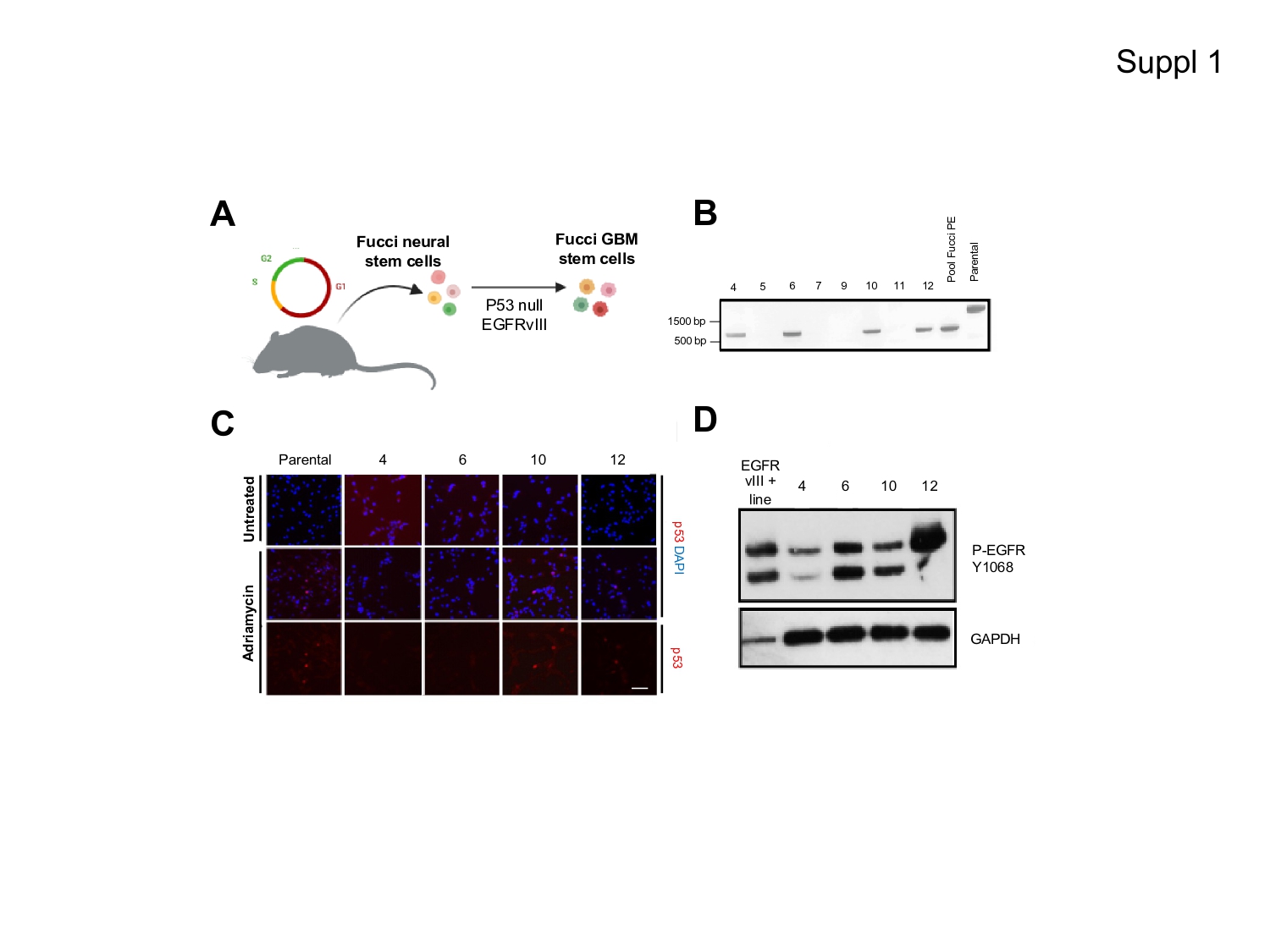

Supplement: Supplementary file 6 [file Image1.jpg]
